# A qualitative study on the perspectives of mothers who had been diagnosed with primary carnitine deficiency through newborn screening of their child

**DOI:** 10.1186/s13023-023-02735-0

**Published:** 2023-06-02

**Authors:** Lieke M. van den Heuvel, Adriana Kater-Kuipers, Tessa van Dijk, Loek L. Crefcoeur, Gepke Visser, Mirjam Langeveld, Lidewij Henneman

**Affiliations:** 1 Department of Human Genetics, Amsterdam UMC, location Vrije Universiteit, Amsterdam, the Netherlands; 2 Amsterdam Reproduction and Development research institute, Amsterdam, the Netherlands; 3 Wilhelmina Children’s Hospital, Section Metabolic Diseases, University Medical Centre Utrecht, Utrecht, the Netherlands; 4 Department of Endocrinology and Metabolism, Amsterdam UMC, location University of Amsterdam, Amsterdam, the Netherlands

**Keywords:** Newborn screening, Maternal primary carnitine deficiency, OCTN2 deficiency, Experience, Opinion, Mothers, Psychological impact, Emotions, Interviews

## Abstract

**Background:**

Primary carnitine deficiency is an inborn error of metabolism, which can lead to life-threating complications early in life. Low carnitine levels can be detected by newborn bloodspot screening (NBS). However, NBS can also identify, mostly asymptomatic, mothers with primary carnitine deficiency. To identify mothers’ needs and areas for improving screening practice, this study explored the experiences with, and opinions on primary carnitine deficiency screening in NBS among women diagnosed through NBS of their newborn.

**Methods:**

Twelve Dutch women were interviewed, 3–11 years after diagnosis. Data were analysed using a thematic approach.

**Results:**

Four main themes were derived: 1) psychological impact of primary carnitine deficiency diagnosis, 2) becoming a patient and “patient-in-waiting”, 3) information issues and care provision, and 4) primary carnitine deficiency as part of the NBS panel. Mothers shared that they did not experience major psychological distress of the diagnosis. They did experience (recall) various emotions following the initial abnormal NBS result, including fear and anxiety as well as relief, and emotions regarding their own diagnosis, including uncertainty about health risks and treatment effectiveness. Some felt a patient-in-waiting. Many participants experienced a lack of information, especially shortly after receiving the abnormal NBS result. All shared the belief that screening for primary carnitine deficiency in NBS is beneficial for the newborn, and, given the information they received, also considered the knowledge beneficial for their own health.

**Conclusions:**

Psychological burden following diagnosis was experienced by women as limited, although the experienced lack of information amplified feelings of uncertainty and anxiety. Most mothers believed that benefits of knowing about primary carnitine deficiency outweighed the disadvantages. Mothers’ perspectives should be incorporated in policy-making about primary carnitine deficiency in NBS.

**Supplementary Information:**

The online version contains supplementary material available at 10.1186/s13023-023-02735-0.

## Background

Primary carnitine deficiency (OCTN2 deficiency, OMIM #212,140) is an inborn error of metabolism, in which low carnitine levels result in impairment of oxidation of long chain fatty acids. Birth prevalence has been reported to be 1 in 30,000-142,000 [1]. It is caused by bi-allelic pathogenic variants in the *SLC22A5* gene [2]. Phenotypic variability is high, even within the same family [3]. Severely affected patients may develop hypoglycaemia, hepatic encephalopathy, cardiomyopathy and/or cardiac arrhythmia that can result in sudden death, while other patients are asymptomatic. Symptoms can be prevented with lifelong carnitine supplementation [3].

With the use of tandem mass spectrometry (MS/MS) technology, it is possible to detect low free carnitine levels in neonatal bloodspot screening (NBS), and hence identify primary carnitine deficiency in newborns [1, 4], enabling early treatment. In many instances, however, the reduced carnitine level is not caused by primary carnitine deficiency in the child, but in the mother [5]. Studies have shown that in this way more mothers than children are identified [4, 6]. In the Netherlands, primary carnitine deficiency is reported in the NBS program as an incidental finding since 2007. In 2015, the Health Council of the Netherlands advised to include carnitine deficiency in the NBS panel, because of the health benefits for newborns [7], however, thus far, the decision to officially include OCTN2 deficiency in the NBS has not yet been made.

Based on the screening principles of Wilson and Jungner [8], NBS should, among other criteria, only include conditions that pose a serious and treatable health problem for the child. Moreover, the benefits of early detection of disorders should outweigh the harms [9]. In clinical practice, it is unknown what kind of follow-up and treatment is needed for, often asymptomatic, women who are identified with primary carnitine deficiency after NBS. Since adult-onset of severe symptoms in asymptomatic individuals is rarely reported, and may affect only a small subset of this population, treatment of all individuals in this group may not be considered necessary [6, 10]. It is therefore debatable whether the benefits of screening for primary carnitine deficiency for the newborns outweigh the disadvantages of detecting asymptomatic women, exposing them to uncertainty and potentially unnecessary treatment and medical follow-up [6, 11]. In New Zealand, in 2017 screening for primary carnitine deficiency was discontinued because of insufficient insight in the benefit of versus harm done by newborn screening [6]. However, even if policy-makers decide that primary carnitine deficiency does not meet the criteria to be formally part of NBS, it still will likely be incidentally detected by most NBS programs that include a range of inherited metabolic diseases identified on acylcarnitine profile in their panels.

Besides knowledge on the medical consequences of primary carnitine deficiency diagnosis and treatment, insight into the perception of women on this diagnosis and the psychological burden they experienced is important in deciding on the desirability of screening for primary carnitine deficiency in NBS and can help to identify their needs. Little is known about the experiences of women diagnosed with primary carnitine deficiency as an incidental finding with NBS, and what their opinions are on including primary carnitine deficiency in NBS. This qualitative study aimed to explore the experiences with, and opinions on primary carnitine deficiency screening in NBS among women diagnosed through NBS.

## Methods

### Setting

NBS in the Netherlands is organised by the Dutch National Institute for Public Health and the Environment (RIVM). Parents receive information on NBS, including a brochure, by an obstetric healthcare provider during pregnancy, and prior to collection of the newborn blood spot by a maternity healthcare provider, youth healthcare worker or midwife. Parents are asked to consent for NBS; the program is not mandatory, but only few parents decline participation (participation rate in 2021 was 99.2%, including 25 disorders) [12]. NBS-samples are obtained within 72 to 168 h after birth. Despite a positive advice of the Health Council in 2015 [7], to include primary carnitine (OCTN2) deficiency in the NBS, it is not yet part of the NBS panel [12]. Nevertheless, the free carnitine (C0) level is determined because a possible deficiency makes the acylcarnitine profile unreliable, which may cause that children with other included metabolic diseases in the NBS remain undetected [12]. Primary carnitine deficiency identified as a result of this procedure is considered an incidental finding [7]. In case of a low free carnitine concentration (≤ 5 µmol/L) a repeat sample will be taken within ten days. When the low free carnitine concentration persists, the medical advisor of the RIVM first consults the metabolic physician for advice and thereafter will contact the general practitioner who generally communicates all abnormal NBS results to parents. The newborn will subsequently be referred to a regional metabolic centre for follow-up, within 24 h. As a result of the evaluation at the metabolic centre, the mother of the newborn can be referred to a metabolic specialist when the low carnitine levels in the newborn are caused by a low level in the mother. The referral rate of mothers increased over time as more reports of maternal primary carnitine deficiency cases identified through NBS were published. When severe signs and symptoms are present in the mother (or in her medical history), lifelong carnitine suppletion is proposed and initiated. If the mother is asymptomatic and clinical evaluation reveals no abnormalities, she is counseled about health risks (unknown, but considered to be low), and treatment is also proposed. Some mothers without symptoms choose to take carnitine supplements only during pregnancy or when they are ill. Others choose a treatment regimen of daily carnitine supplementation.

### Design

We used a semi-structured qualitative interview design to capture the range and diversity of (past) experiences and perceptions of mothers diagnosed with primary carnitine deficiency after NBS in their child. The study protocol was reviewed by the Medical Ethical Committee of the Amsterdam UMC, location VU University Medical Center, and deemed exempt from further review because the Act of Medical Research Involving Human Subjects (WMO) did not apply (METC No. 2019 − 509).

### Participants

Participants were recruited via a researcher (L.C.) studying the clinical and biochemical aspects of primary carnitine deficiency in the Netherlands (the ODIN study, Trial number NL7905). The ODIN study enrolled 37 women with confirmed primary carnitine deficiency. During their intake for the ODIN study at one of the five participating centres, women were asked if they were also interested in participating in the interview study. If so, they received an information letter about the study and their contact details were given to the researchers conducting the interviews (A.K. or T.v.D.). Those confirming interest in participation were scheduled for an interview and asked to provide written informed consent.

Nineteen women in the ODIN study were asked to participate in the interview study. Sixteen confirmed interest in study participation, of whom 13 women after contact wanted to participate. In total, 12 women were interviewed. One woman indicating interest eventually refrained from participation. Table 1 shows characteristics of participants. The median age was 36.5 years (IQR = 10). Most participants (75%) had a high education level. The interviews were conducted 3–11 years (Median = 5; IQR = 4) after the primary carnitine deficiency diagnosis. Of the participants, four (33%) currently reported symptoms which they assigned to the primary carnitine deficiency, including fatigue. Eight out of twelve participants (67%) used carnitine supplementation.

**Table 1:** Characteristics of interview participants

Characteristic	N = 12
Age, median (IQR)	36.5 (10)
Married/living together, n (%)	12 (100)
Education level^a^, n (%)	
Low	1 (8)
Moderate	2 (17)
High	9 (75)
Number of children, median (IQR)	2 (1)
Time in years since primary carnitine deficiency diagnosis^b^, median (IQR)	5 (4)
Self-reported symptoms assigned to OCTN2 deficiency^c^, n (%)	4 (33)
Taking medication for primary carnitine deficiency, n (%)	8 (67)

IQR = Interquartile Range.

^a^Low: elementary school, lower level of secondary school, lower vocational training; Moderate: higher level of secondary school, intermediate vocational training; and High: higher vocational training university.

^b^Interviews were conducted between September 2020 and April 2021.

^c^At the time of the interview, i.e., fatigue/lack of energy (n = 3) and muscle weakness (n = 1).

### Data collection

The interview guide was developed based on literature, and in collaboration with a multidisciplinary team consisting of experienced qualitative researchers with expertise in health science, and physicians who monitor women diagnosed with primary carnitine deficiency. The interview guide addressed the experience of an abnormal (false positive) NBS result in their child, the impact and consequences of the diagnosis of primary carnitine deficiency by NBS for themselves, and the perceived pros and cons of screening for primary carnitine deficiency in NBS (see Supplementary Material Appendix A). All interviews were conducted by telephone due to the COVID-19 pandemic. Interviews were performed by A.K. (a social researcher, conducted six interviews) and T.v.D. (a medical researcher, conducted six interviews), who both had prior experience in qualitative research. The interviewers were not involved in the care for these women; participants were informed that the interviewers had no access to their patient record. The interviews were conducted between September 2020 and April 2021 and lasted between 30 and 60 min.

### Data analysis

Interviews were audio-recorded and transcribed verbatim. Thematic analysis was conducted in parallel with interviewing [13]. Two researchers independently (A.K and T.v.D.) and inductively coded the first five interviews, using Atlas.ti software. Differences in coding and findings were discussed until agreement was reached. The remaining transcripts were subsequently coded, and codes were then grouped and ranked into a coding tree, outlining the main themes identified in the interview data. Main themes and subthemes were subsequently discussed with A.K., T.v.D. and L.H., and further refined until agreement. Quotes used in the manuscript were translated in English to illustrate the themes identified. We complied to the COREQ checklist for reporting and analysing qualitative data [14].

## Results

The following themes were derived from the interview data: 1) psychological impact of primary carnitine deficiency diagnosis, 2) becoming a patient and “patient-in-waiting”, 3) information issues and care provision, and 4) primary carnitine deficiency as part of the NBS panel.

### Psychological impact of primary carnitine deficiency diagnosis

Most participants mentioned that having primary carnitine deficiency did not have a huge impact on their psychological functioning in daily life. However, participants did experience several emotions associated with the initial NBS result, the primary carnitine deficiency diagnosis and the follow-up care:

### Fear and anxiety

Participants recalled feeling fearful about their child’s health when being informed initially of the abnormal NBS result. The fact that the general practitioner put urgency to this message and the necessity to go to the hospital for confirmation of findings amplified these emotions as well as raised anxiety. A participant shared her experience:

“*That weekend was really nerve-wrecking, that we thought: okay, our child might be really sick or, what is it exactly? You’ve never heard of it before. And then it became clear pretty quickly: there’s nothing wrong at all. But that first weekend, that was a bit of a shock, I remember*.” (Participant #7)

Nevertheless, most participants understood the urgency put to it, as they believed this could save their child’s life. One participant said she did not experience fear when hearing the abnormal NBS result and intuitively had the idea that their child did not have a severe condition, because he or she did not seem sick. When it became clear that mothers themselves were diagnosed with a medical condition, some participants felt anxious about the possibility to develop severe symptoms because of the carnitine deficiency. A few also expressed their worries about potential implications for family members.

One participant remained worried about her child having carnitine deficiency, despite the fact that she was informed that the child did not have the condition:

*“You always think, if she [daughter] is tired or having the flue…is this it [carnitine deficiency]? Is she not too lethargic? Everything is associated with it. That feeling will never disappear.”* (Participant #2)

### Relief

When it turned out that participants themselves, instead of their child, had primary carnitine deficiency based on the abnormal NBS result, all participants experienced relief regarding the health of their child, especially when participants themselves did not experience complaints at the time of diagnosis. A participant recalled:

“*I worried a lot about my child [after the positive NBS result], I noticed afterwards, and that affected me a lot. But that was more the uncertainty [about the meaning of the positive NBS result]. And I thought to myself: I’m doing well, and I feel good, so I don’t think it’s very serious*.” (Participant #10)

Some asymptomatic participants also felt relieved that, based on the information they received at the hospital, it did not seem a life-threatening condition they were diagnosed with. Others considered the diagnosis an explanation for symptoms they experienced, sometimes for many years. These mothers were relieved that a potential explanation was found. One participant said:

“*It was actually, maybe also sort of nice that it [symptoms] was confirmed, because it was of course always a search for ‘what’s wrong with me’? And nothing ever came out of it. So yes, at a certain point you think: maybe it’s all in my head or something like that*.” (Participant #8).

### Uncertainty and disbelief

At the time of diagnosis, some participants said they never had symptoms that could be related to carnitine deficiency and therefore they could not believe that they really had a condition or that it could be(come) a severe disease. Especially for asymptomatic participants it felt strange: diagnostic tests were performed and treatment was indicated for a condition of which they perceived no symptoms. During the interviews, two participants shared that they still experienced uncertainty regarding potential symptoms of the condition in the future.

Participants felt that lack of information provision amplified the feeling of uncertainty. For some, the uncertainty made them also question the diagnosis of the condition: how is it possible to have a disease while they did and do not have any symptoms? Also some were puzzled about the inheritance, causing confusion:

*“It’s almost kind of implausible, because my parents should both have the wrong gene and you think: well, that’s not possible at all, is it [because they did not have any symptoms either]? So you also sort of think: is it all really true? Because I didn’t notice anything [any symptoms] before that [diagnosis].”* (Participant #2)

### Becoming a patient and “patient-in-waiting”

Multiple participants said they felt they had become “a patient” because of the diagnosis, although they had no or only very mild symptoms. They felt like they were waiting for the moment they would develop symptoms. One participant explained that knowing about the condition while not having any symptoms felt like a potential threat. She said:

“*If you know that you can do something about it, then you have something to hold on to. [But] as long as you have no complaints, and you do know that you have something, then it keeps circling around somewhere above you. In that way it could go in all kind of directions but, yeah, there is not much you can do about it.”* (Participant #2).

The idea of clinical monitoring and having to take lifelong medication to prevent getting sick was by some described as burdening. A participant explained:

“*I do remember the first time I went to the general practitioner to get the medication. Well, they gave me a large box of bottles and that was for only one month. And then I thought: now I really am seriously ill. I thought this is going to go on for the rest of my life, I just have to have a box of that stuff. So yeah, that was really impactful*.” (Participant #4)

Some shared the feeling that healthcare professionals made them a patient, as the medical specialist advised treatment for something the participants did not have symptoms of.

Participants recalled that taking medication was presented as the right thing to do. Some did not even recall that the pros and cons of the medication were discussed with them. A participant said:

“*Was I really asked if I wanted to take medication or not? I don’t really remember. I don’t know if I was really asked to choose. I don’t think so. And if I really didn’t want to, I could have said so, of course*.” (Participant #3)

Four participants shared that they started with the medication but stopped after some time, because they did not perceive the usefulness of taking it because they were asymptomatic or they felt it did not help feeling better and/or due to side effects (i.e. medication-related stomach pain (n = 1), unpleasant smell of urine and sweat (n = 1)). Two women who took carnitine supplementation also mentioned the unpleasant smell of urine and one woman questioned the costs associated with taking the medication. In contrast, one participant said taking the medication reassured her since she felt that it would prevent the development of severe symptoms (such as arrhythmias), although she is currently asymptomatic.

### Information issues and care provision

#### Information prior to NBS

Prior to NBS, none of the participants had thought of, or heard about the possibility that NBS could also reveal something about their own health, instead of the newborn’s health. Some participants believed that information provision prior to newborn screening should therefore entail that the result of NBS could also have implications for the mother (which is currently not addressed in the parental information before NBS). In contrast, it was also mentioned that this could cause “unnecessary” anxiety, and therefore should perhaps not be included in information provision about NBS.

#### Information during the diagnostic process

Participants stressed the importance of adequate information provision, particularly shortly after receiving the abnormal NBS result by the general practitioner. They reported that the information they received was very limited. Participants missed information about what primary carnitine deficiency exactly is and what they could expect regarding symptoms. The vagueness regarding the consequences of the disorder in general and the diagnostic process raised fears regarding the health of their newborn. It was also mentioned that it would have helped them right at that moment of receiving NBS results to hear that it could also be a consequence of the mother’s health, as these participants thought they would have worried less. A participant explained:

“*Well, it could have been a bit calmer, maybe a bit more information, a bit less like: ‘You have to go to the hospital now and see the emergency doctor. ... And maybe a side note that it didn’t necessarily mean that she [daughter] had it, because it was, let’s say, it was then presented as a fait accompli that she would have it. And I only found out later that it was also possible that she didn’t have it, but that I, for example, had it or something*.” (Participant #11)

In general, participants were positive about the diagnostic follow-up process. They felt that the consultation and diagnostic tests at the hospital did not last long and brought more information, and in the end clarity about their child’s health. Two participants had however negative memories about the diagnostic process and experienced concerns and anxiety when their newborn had invasive diagnostic tests:

“*But then... the ball started rolling, so she [the child] had to be tested (…), a one-week-old baby getting terrible needles in her body, yes, I found that really, really intense*.” (Participant #6)

A few participants recalled that their newborn also received medication directly after the abnormal NBS result of primary carnitine deficiency. These participants expressed doubts about this, as they themselves instead of their child could be carnitine deficient and were worried that the child had been given unnecessary, and possibly harmful, medication. A participant described her feeling:

“*I felt like: if it turns out that it’s only me, then we’re going to give the baby medication now, which may not be necessary at all, and isn’t that harmful?... That was the only thing that bothered me all the time. Like, OK, well, we’ll give her medication now, and you don’t actually have the results of the test yet. I found that very annoying*.” (Participant #5)

#### Long-term follow-up care of mothers

Participants generally had the experience that healthcare professionals, including general practitioners and paediatricians, seemed not to have sufficient knowledge on primary carnitine deficiency in general, and specifically when diagnosed in mothers through NBS. It was mentioned that information received by healthcare professionals who were not specialized in primary carnitine deficiency, was lacking or even wrong. Many participants thought that such information was just unknown, and that scientific research was lacking. A participant for example said:

“*That is also the difficult part: there is still so much unknown [about primary carnitine deficiency], so I am curious about the results of these studies [Dutch research project, ODIN study]. Also, is it logical that the fatigue is caused by that [primary carnitine deficiency] or not? And then of course mainly, what does it matter, for my own risk on adverse disease symptoms.“* (Participant #12)

### Primary carnitine deficiency as part of the NBS panel

All women were positive about the possibility to detect primary carnitine deficiency by NBS and believed primary carnitine deficiency in the newborn and/or the mother should be reported to parents. They considered it important to know for their child’s health, although they eventually appeared not affected. Most also believed that knowledge about having primary carnitine deficiency themselves was a good thing, as they explained that they now know why they have certain symptoms and/or are enabled to prevent possible future symptoms. Participants also said that being diagnosed with primary carnitine deficiency would be helpful in case physical complaints associated with primary carnitine deficiency arise in the future. A participant mentioned:

“*Yes, I think: if something happens to me and they do know about it [primary carnitine deficiency], then they can at least make sure that it stays that way and that it’s good, that my body doesn’t have to work harder because of it or something*.” (Participant #9)

Two participants had doubts about the benefits of knowing for themselves: they said that it is not something they can easily cope with, as the information they receive by healthcare professionals about primary carnitine deficiency in terms of associated symptoms and effectiveness of treatment keeps being vague. They, however, still believed that it should be reported, either as incidental finding or as targeted disorder in NBS. Others clearly indicated that they believed it should definitely be integrated in the NBS panel, and not be reported as incidental finding; they thought the benefits of knowing about a potential severe illness of their child but also their own diagnosis outweighed the disadvantages of it.

## Discussion

This study describes the experiences with and impact of primary carnitine deficiency as reported by women diagnosed with the condition as a result of NBS in their newborn. The women in this study shared that they did not experience high levels of (long-term) psychological burden being diagnosed with primary carnitine deficiency, although they reported various feelings and emotions throughout the diagnostic and follow-up process. Some felt a patient-in-waiting. A lack of information provision was generally reported, and also experiencing a lack of knowledge on primary carnitine deficiency among healthcare professionals. Despite these experiences, all women believed that benefits of knowing about primary carnitine deficiency for both the newborn as well as themselves outweighed the disadvantages, and therefore believed it should be included in NBS.

Most women interviewed in this study did not experience a huge impact of the primary carnitine deficiency diagnosis through NBS on psychological functioning in daily life. This finding contrasts to findings of a large cohort study in Germany described by Schiergens et al. [11], who stated that several women diagnosed with primary carnitine deficiency through NBS in their newborn reported high psychological burden, due to the unclear clinical risk of the primary carnitine deficiency. There are, to our knowledge, no other studies reporting on psychological impact of screening for primary carnitine deficiency in NBS. Although psychological impact was experienced as limited by most women in our study, they did experience a wide range of emotions related to the initial NBS result and diagnosis, including fear and anxiety towards the potential severe diagnosis in their newborn but also relief that their newborn appeared to be healthy, uncertainty about the implications for themselves, and, for those experiencing symptoms, to have an explanation for their experienced symptoms. Ongoing worry about the child’s health was reported by one participant, which is a well-known phenomenon seen in parents receiving a false positive result [15]. Some women also expressed the feeling that the diagnosis made them a patient, while they had not experienced any symptoms. Timmermans and Buchbinder [16] referred to such patients as ‘patients-in-waiting’, defined as ‘people trapped between a state of sickness and health, characterised by uncertainty about disease’. It is important that healthcare professionals involved are aware of these issues during the diagnostic and follow-up process to enable mothers to share these feelings and discuss their needs in this regard.

The uncertainty women in this study experienced was also related to the feeling that healthcare professionals were not very knowledgeable about primary carnitine deficiency, both about the diagnosis and the follow-up care. Participants experienced a lack of information provision and knowledge from the general practitioner reporting the abnormal results and from health professionals making the diagnosis. This corresponds to experiences of parents participating in NBS who received an abnormal result for other disorders [17], and in other countries [18, 19]. This may indicate a lack of consistency in healthcare professionals and strategies used to disclose NBS results [17–19]. In addition, women in our study reported a lack of knowledge among healthcare professionals, including medical specialists, on associated symptoms and treatment effectiveness after diagnosis of primary carnitine deficiency, amplifying feelings of uncertainty and anxiety. However, some participants also thought that there is just not enough knowledge (yet) on this subject. A need for improved information provision, including development of written information, in different stages of the diagnostic and follow-up process was expressed as a mean to better support these women and to diminish uncertainty and anxiety. In the literature, the importance of early involvement of specialists, continued monitoring by trusted providers and psychological and social support was also stressed to reduce parental anxiety after abnormal NBS results [15]. Importantly, women were generally positive about primary carnitine deficiency being part of NBS, because of the expected positive outcome for the newborn but, for many women, also because of potential perceived benefits for their own health given the information they received regarding treatment effectiveness. Questions about the exact benefits of knowing about the diagnosis for themselves were, however, also raised. As stated in the Wilson and Junger principles [8], the potential benefit from the screening program should outweigh potential harm, both physical and psychological harm. To determine physical and psychological harm, experiences and attitudes of women diagnosed are important to incorporate in decision-making on including primary carnitine deficiency in NBS [20].

### Strengths and limitations

To our knowledge, this study is one of the first to explore the experiences and opinions of women diagnosed with primary carnitine deficiency through NBS. Our findings provide valuable insights for healthcare professionals and policy-makers deciding on screening for primary carnitine deficiency in NBS. However, this study also had some limitations. Due to the rarity of this diagnosis, we could only conduct a relatively small number of interviews, with most women being highly educated. Future research should include experiences of less educated and less integrated women with difficulties understanding Dutch. Due to COVID-19, the interviews were conducted by telephone instead of face-to-face interviews; non-verbal communications could therefore not be observed. Finally, the interviews were focused on current experiences and attitudes, but also on experiences in the past. In most cases, the diagnosis was made several years before the interview, and it is likely that incorrect recall and/or selective retention of information and attenuation of the intensity of the initial psychological reaction occurred. Moreover, regarding women’s experienced lack of information provision; we do not know whether the information was given or whether women did not retain the information. Nevertheless, the study highlights several important issues from a mother’s perspective.

## Conclusions

This qualitative study describes the experiences of women diagnosed with primary carnitine deficiency through NBS. While women generally reported that they experienced the psychological impact of the diagnosis and follow-up as limited, they experienced a wide range of emotions, including being a patient-in-waiting and the uncertainty associated with this. Given the information women received, most believed that benefits of knowing about primary carnitine deficiency outweighed the disadvantages and therefore believed screening for this should be continued. Healthcare professionals involved in the care for these women should be aware of the feelings and emotions that may exist among mothers confronted with such a diagnosis. In addition, information provision -both the content of information and the way to provide it- early after the NBS result, but also during follow-up care should be improved. Research on the chance on developing symptoms as well as treatment effectiveness is needed to better inform women diagnosed with primary carnitine deficiency through NBS. The findings of this study give insight into the experiences and opinions of mothers diagnosed through NBS. It is important that perspectives of stakeholders involved, including mothers, are incorporated in decision-making on NBS [20].

## Electronic supplementary material

Below is the link to the electronic supplementary material.

Supplementary Material 1

## Data Availability

Data generated or analysed during this study are included in this published article (and its supplementary information files). The transcripts are not publicly available due to the privacy of the research participants.
