# Supplementary material for: A qualitative study on the perspectives of mothers who had been diagnosed with primary carnitine deficiency through newborn screening of their child
Source: Orphanet J Rare Dis. 2023 Jun 2;18:134. doi: 10.1186/s13023-023-02735-0 (PMC10236393; doi:10.1186/s13023-023-02735-0)
Supplement: Supplementary file 1 — Supplementary Material 1 [file 13023_2023_2735_MOESM1_ESM.docx]

**Supplementary Appendix A**

**Interview guide for the study:**

*van den Heuvel et al. A qualitative study on the perspectives of mothers who had been diagnosed with primary carnitine deficiency through newborn screening of their child.*

Thank you for participating in this interview.

**Brief explanation about the study:**

A study to determine whether OCTN2 deficiency should and could be added to the heel prick is currently being commissioned by the Ministry of Health, Welfare and Sport and the RIVM. In the Netherlands, all children are offered the heel prick shortly after birth. The heel prick involves taking some blood from a child's heel. A laboratory tests this blood for a number of severe, rare diseases. The diseases can be treated, for example with drugs or diet. By detecting the diseases early, treatment can start quickly. This prevents serious damage to a child’s health and development.

An abnormal result for primary carnitine deficiency, OCTN2, is currently still considered an incidental finding from the heel prick. This means that it is not intended to detect this disease, but there is still a small chance that the heel prick result is abnormal for OCTN2. If the heel prick result is abnormal for OCTN2, follow-up testing at the hospital may reveal that not the child, but the mother has OCTN2 deficiency.

Two investigations are carried out nationwide. First, women diagnosed with OCTN2 deficiency after their child's heel prick will have some clinical data collected to see how the disease progresses. You have already been contacted for this study (the ODIN study). Secondly, interviews will be used to explore what it means for these women to have this diagnosis. That is what this study is about. By conducting personal interviews, we want to understand what the consequences of this diagnosis and possible treatment are for women like you. We are also curious to know how you look back on the heel prick result. In this way we hope to improve the heel prick screening and the information about it if necessary.

**Experience with heel prick and diagnosis of OCTN2 deficiency**

1. How long has it been since the heel prick was taken from your child (birth)?
2. You were told at the time that the heel prick was abnormal. Can you tell us more about that?
   - How did it proceed?
   - How did you experience it?
3. When did it become clear to you that you had a metabolic condition and not your child?
   - How did that happen?
   - What was your first reaction to this?

- What was the result like for you? How did you deal with it?
- Had you thought about this possibility beforehand?
- Looking back on this, would you have liked to be informed about this possible result beforehand? What information and why?

**Information, treatment and impact**

1. How did you proceed after being diagnosed with OCTN2 deficiency?
2. Who gave you the information about OCTN2 deficiency?

- How did you feel about it/do you feel about it?
- What information did you receive after you were diagnosed with OCTN2 deficiency?
  - - How did you find that?

1. Did you seek any further information?
   - What, why and where?
2. Are you currently under treatment for OCTN2 deficiency?

- What does that treatment consist of? What is it like for you?

1. What does the diagnosis of OCTN2 deficiency mean for you personally?

- How does it affect your daily life?
- Do you experience limitations or symptoms, and if so, which ones? Can you give an example of these?

1. What advantages does it have for you to know that you have OCTN2 deficiency?
2. What disadvantages does it have for you to know that you have OCTN2 deficiency?
3. When you weigh up these advantages and disadvantages, would you have wanted to know that you have OCTN2 deficiency? Why (not)?
   - How do you think other mothers (parents) feel about this?
4. To what extent do you ever worry about your health as a result of OCTN2 deficiency?
   - Can you tell a little more about this?
5. To what extent do you worry about the health of your child or other family members?
   - Can you give an example of this?
6. Did you have more children or do you still have a desire to have children? And to what extent does the diagnosis of OCTN2 deficiency play a role in this?
   - Can you tell a little more about this?

**OCTN2-deficiency in heel prick screening**

Explanation: Since 2007, OCNT2 deficiency has been detected as an incidental finding with the heel prick. This means that OCNT2 deficiency is not specifically looked for, but it is identified when screening for other diseases. Also - as you know - occasionally mothers (without symptoms) are found instead of children with OCTN2. However, because OCTN2 deficiency can present as a serious metabolic disease in newborns and good treatment is available, the Health Council recommended in 2015 that this condition should also be officially included as a target disease in heel prick screening.

1. What do you think of this advice?
2. Do you think OCTN2 deficiency is a condition that belongs in heel prick screening?
   - Why yes/no?
3. Do you think the information around heel prick screening should be updated if OCTN2 is formally added to the screening?

- How?
